# Influence mechanism of serum free immunoglobulin light chain on pulmonary inflammatory response and serum levels of inflammatory factors in patients with chronic obstructive pulmonary disease

**DOI:** 10.5937/jomb0-49201

**Published:** 2024-11-16

**Authors:** Ying Fang, Dandan Hu, Qin Li, Mei Chen, Cuiying Yin

**Affiliations:** 1 Third Affiliated Hospital of Zhejiang Chinese Medicine University, Department of Respiratory and Critical Care Medicine, Hangzhou, Zhejiang Province, China; 2 Third Affiliated Hospital of Zhejiang Chinese Medicine University, 2Department of Critical Care Medicine, Hangzhou, Zhejiang Province, China

**Keywords:** chronic obstructive pulmonary disease, serum free immunoglobulin light chain, inflammatory factors, lung function index, hronična opstruktivna bolest pluća, laki lanac imunoglobulina u serumu, upalni faktori, indeks funkcije pluća

## Abstract

**Background:**

The pathogenesis of chronic obstructive pulmonary disease (COPD) is not fully understood. This work aimed to demonstrate the role of serum free light chains (sFLC) in the pulmonary inflammatory response of COPD patients and its relationship with serum inflammatory cytokine (IC) levels.

**Methods:**

Eighty subjects were enrolled, including 40 COPD patients (COPD group) and 40 healthy controls (control group). All patients were further rolled into four subgroups regarding the Global Initiative for Chronic Obstructive Lung Disease (GOLD) staging criteria. The serum levels of sFLC and ICs were compared between the two groups, and their correlations with lung function indicators were analyzed.

**Results:**

The serum sFLC levels of COPD patients were markedly superior to those of healthy controls. The levels of serum sFLC and ICs (tumour necrosis factor-a (TNF-a), interleukin (IL)-6, IL-8) gradually increased with the severity of the disease. The levels of ICs were negatively correlated with lung function indicators (FEV1% predicted, FEV1/FVC).

**Conclusions:**

These findings suggest that serum sFLC may play a critical role in the pulmonary inflammatory response of COPD patients and serve as a potential indicator for evaluating COPD severity and predicting disease progression.

## Abstract (sr)

**Uvod:**

Patogeneza hronične opstruktivne bolesti pluća (HOPB) nije potpuno razjašnjena. Ovaj rad ima za cilj da prikaže ulogu slobodnih lakih lanaca u serumu (sFLC) u plućnoj inflamatornoj reakciji kod pacijenata sa HOPB i njihov odnos sa nivoom upalnih citokina u serumu.

**Metode:**

Uključeno je osamdeset ispitanika, od kojih 40 pacijenata sa HOPB (grupa HOPB) i 40 zdravih kontrolnih pacijenata (kontrolna grupa). Svi pacijenti su dalje podeljeni u četiri podgrupe u skladu sa kriterijumima za klasifikaciju Globalne inicijative za hroničnu opstruktivnu bolest pluća (GOLD). Upoređivani su nivoi sFLC-a i IC-a u serumu kod ove dve grupe i analizirana je njihova korelacija sa indikatorima plućne funkcije.

**Rezultati:**

Nivoi sFLC-a u serumu kod pacijenata sa HOPB su bili značajno viši u poređenju sa zdravim pacijentima. Nivoi sFLC-a i IC-a (faktor nekroze tumora-a (TNF-a), interleukin (IL)-6, IL-8) u serumu su postepeno rasli sa težinom bolesti. Nivoi IC-a su bili u korelaciji sa indikatorima plućne funkcije (FEV1% predviđeno, FEV1/FVC).

**Zaključak:**

Ovi rezultati ukazuju da sFLC u serumu može da ima ključnu ulogu u plućnoj inflamatornoj reakciji kod pacijenata sa HOPB i može da posluži kao potencijalni indikator za procenu ozbiljnosti HOPB i predviđanje progresije bolesti.

## Introduction

Chronic obstructive pulmonary disease (COPD) is characterized by airway inflammation, airflow limitation, and alveolar destruction. The major causes of COPD include smoking, long-term exposure to harmful environmental factors (such as air pollution and occupational exposure), and genetic factors [1] [2]. Globally, COPD has become one of the leading disease burdens, severely affecting the life expectancy of patients. The clinical manifestations of COPD mainly include persistent cough, sputum production, shortness of breath, and reduced exercise tolerance. Its diagnosis relies primarily on pulmonary function tests, especially by evaluating the ratio of forced expiratory volume in one second (FEV1) to forced vital capacity (FVC) to assess the degree of airway obstruction. COPD patients may also experience acute exacerbations, which manifest as a significant worsening of symptoms and require prompt treatment [3] [4] [5].

The pulmonary inflammatory response in patients with COPD plays a crucial role in pathological and physiological processes. Prolonged exposure to harmful environmental factors can damage airway epithelial cells, alveolar cells, and immune cells (such as macrophages and neutrophils), triggering an inflammatory response [6]. In addition, inflammatory mediators by inflammatory cells, such as tumour necrosis factor-α (TNF-α), interleukin IL-6, and IL-8, can further promote the persistence and exacerbation of inflammation [7] [8]. Chronic airway inflammation leads to airway remodeling and narrowing and causes pathological changes such as the thickening of airway smooth muscles and enlargement of mucous glands [9]. Alveolar destruction, airway inflammation, and reduced lung function are typical manifestations of COPD, which make patients prone to symptoms such as dyspnea and shortness of breath. Since chronic inflammation plays a core role in the course of COPD, intervention in inflammation is of great clinical significance [10] [11] [12].

Immunoglobulin (Ig) is a protein that acts in the immune response and is composed of heavy and light chains. Excess light chains are produced during immunoglobulins synthesis, forming free light chains (FLC). Serum FLCs are mainly composed of two types, kappa (κ) and lambda (λ), and participate in physiological processes such as immune regulation, inflammation, and cell proliferation [13] [14] [15] [16]. In recent years, it has been found that sFLCs show abnormal elevation in many inflammatory diseases, such as autoimmune diseases, infectious diseases, and chronic inflammatory diseases. Therefore, sFLCs are considered a novel inflammation and immune regulatory factor, and their role and mechanism in patients with COPD are worth further investigation [17] [18].

This work aimed to demonstrate the changes in sFLC in COPD patients and their effects on pulmonary inflammation and serum inflammatory cytokine (IC) levels in COPD patients. Through this work, we hope to gain a deeper understanding of the mechanisms of pulmonary inflammation in COPD patients and provide new therapeutic strategies for clinical practice. In addition, this work may also provide valuable references for the diagnosis and treatment of other inflammatory diseases.

The novelty of this study lies in its exploration of sFLC as potential biomarkers for the pulmonary inflammatory response in patients with COPD and their association with serum inflammatory cytokine (IC) levels. Additionally, studying the correlation between sFLC levels and lung function indicators could show their potential utility as biomarkers for evaluating COPD severity and predicting disease progression. This aspect of the study contributes to the field by proposing a new avenue for understanding and managing COPD, potentially leading to more targeted therapeutic interventions and personalized treatment strategies.

## Materials and methods

### Study population and group allocation

Eighty patients were included, including 40 diagnosed with COPD (COPD group) and 40 healthy controls. According to the Global Initiative for Chronic Obstructive Lung Disease (GOLD) guidelines, the COPD group patients all met the diagnostic and grading criteria for COPD. The inclusion and exclusion criteria for the study are presented in Table 1 and Table 2, respectively.

**Table 1:** Inclusion criteria.

No.	Inclusion criteria
1	Subjects who met the criteria of GOLD for COPD
2	The age was between 40 and 75, regardless of sex
3	Patients without acute exacerbation symptoms
4	Patients or their families agreed to participate in this work and sign the informed consent form

**Table 2:** Exclusion criteria.

No.	Exclusion criteria
1	Patients with other respiratory diseases (such as bronchial asthma and pulmonary fibrosis)
2	Patients with severe cardiovascular, liver and kidney dysfunction, or malignant tumor
3	Pregnant and lactating women
4	Patients who had received immunosuppressive treatment in the past three months.
5	Patients with infectious diseases in the near future (within one month)
6	Patients who refused to participate in the study or could not cooperate to complete the study.

The study subjects were rolled into COPD and the healthy control groups. The COPD group was further assigned into four subgroups based on the GOLD grading criteria, including mild (GOLD 1), moderate (GOLD 2), severe (GOLD 3), and very severe (GOLD 4) subgroups. The healthy controls were the recruited volunteers who matched the baseline characteristics of COPD patients, including age, sex, and body mass index (BMI). The controls must have no history of respiratory, cardiovascular, liver or kidney dysfunction or other major illnesses. Basic information of all study subjects should be recorded, including age, sex, height, weight, smoking history, allergy history, and family history. Meanwhile, clinical information related to COPD, such as course of disease, complications, and treatment, was also collected.

### Specimen collection and processing

Blood, sputum, and lung tissue samples were collected from the participants. Venous blood (approximately 5 mL) was collected from fasting subjects, with 2 mL used for routine blood testing and 3 mL for serum separation. Serum samples were processed by centrifugation and stored at -80°C. Sputum samples were collected through non-stimulated coughing and filtered to remove impurities before freezing. Lung tissue samples were obtained from COPD patients undergoing lung biopsy or resection, divided for histological and biochemical analyses, and stored at -80°C.

### Immunohistochemical and ELISA methodologies

Immunohistochemical analysis was conducted on lung tissue samples to qualitatively and quantitatively assess inflammatory cytokines (ICs) and immunoglobulin light chains. Paraffin-embedded tissue sections were processed and stained with specific primary antibodies against TNF-α, IL-6, IL-8, and immunoglobulin light chains κ and λ. Fluorescently labelled secondary antibodies were applied, and images were captured and analyzed. Enzyme-linked immunosorbent assay (ELISA) was used to quantify ICs (TNF-α, IL-6, IL-8) and free immunoglobulin light chains (FIGLCs) (κ and λ) in serum and sputum samples. Standard curves were established, and absorbance was measured using an ELISA reader.

### Bradford protein quantitative determination

Total protein content in frozen lung tissue samples was determined using the Bradford method. Thawed samples were treated with a protein extraction buffer, and protein content was quantified using a spectrophotometer based on a standard curve.

### Statistical methodologies

Descriptive and inferential analyses were performed on the data. For continuous variables, mean±standard deviation or median with interquartile range was reported based on data distribution. Categorical variables were described using frequency and percentage. Statistical tests, including t-tests, Mann-Whitney U tests, Chi-square tests, Fisher’s exact tests, Pearson correlation coefficient, and Spearman correlation coefficient, were applied as appropriate for comparisons and correlation analyses.

## Results

### Demographic characteristics of subjects

This work included 80 patients, including 40 diagnosed with COPD (COPD group) and 40 healthy controls. The baseline characteristics of the two groups of patients included age, sex, height, weight, smoking history, allergy history, family history, duration of illness, comorbidities, and treatment. Compared by independent sample t-test and chi-square test, the results suggested inconsiderable differences in baseline characteristics between the two groups (P>0.05), indicating comparability. The specific data is presented in Table 3.

**Table 3:** Demographic characteristics of subjects included into the study.

Variables	COPD group<br>(n=40)	Control group<br>(n=40)	*P*
Age (years)	62.3±7.6	63.1±8.2	0.658
Gender (M/F)	24/16	22/18	0.478
Height (cm)	170.4±8.5	171.2±7.8	0.572
Weight (kg)	68.3±10.2	69.1±9.6	0.653
Smoking history<br>(Y/N)	20/20	18/22	0.605
Allergy history<br>(Y/N)	5/35	4/36	0.725
Family history<br>(Y/N)	8/32	7/33	0.788
Disease duration<br>(years)	5.2±3.1	-	

### Effect of serum FIGLC on pulmonary inflammatory response in patients with COPD

**Figure 1:**
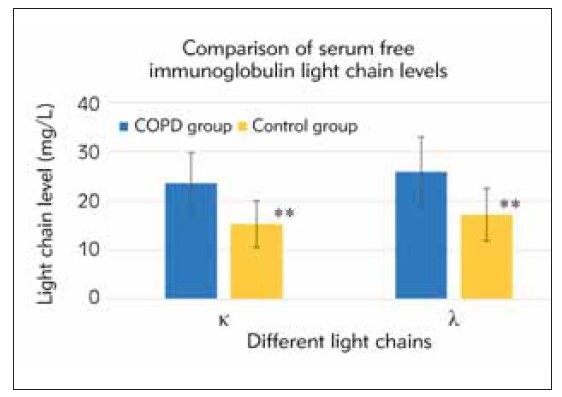
Comparison of serum FIGLC levels. Note: **P<0.05 between two groups.

ELISA detected serum levels of FIGLCs (κ and λ), and the differences between the two groups were compared. The results indicated that the serum levels of FIGLCs in the COPD group were markedly superior to those in the healthy controls (P<0.05). The specific data is presented in Figure 1.

**Figure 2:**
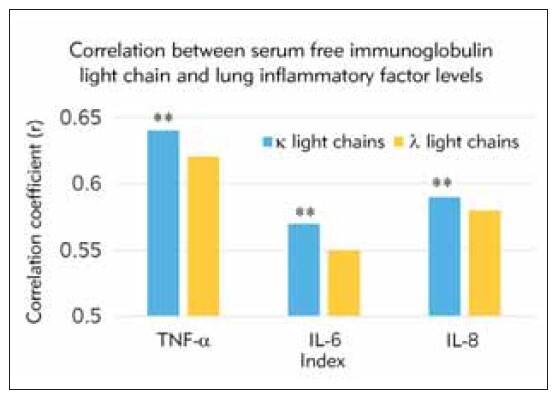
Correlation between serum FIGLC and pulmonary IF level. Note: **P<0.05 between the two kinds of serum FIGLCs

Furthermore, the relationship between pulmonary inflammatory factors (IF) (such as TNF-α, IL-6, and IL-8) and serum FIGLCs in the COPD group was further analyzed. The results demonstrated a positive correlation between serum levels of FIGLCs and pulmonary IF levels (P<0.05). The specific correlation coefficients are presented in Figure 2.

### Comparison of serum FIGLC and IFs in patients with various GOLD grades

**Figure 3:**
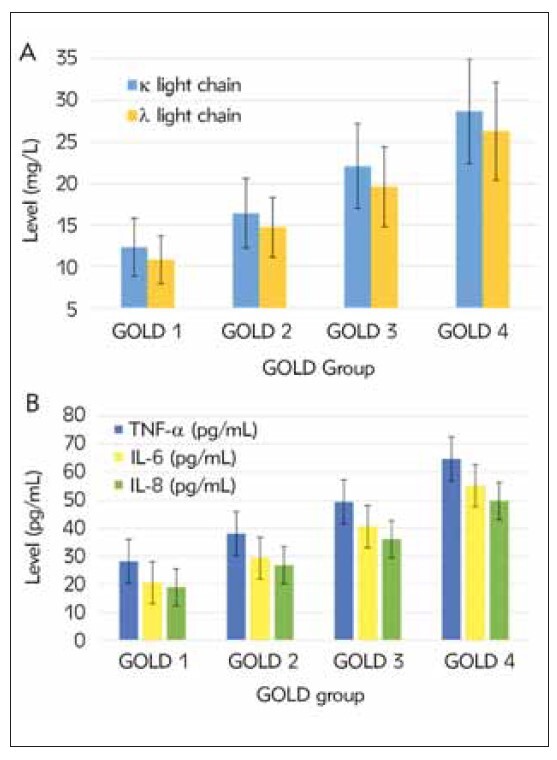
Comparison of serum FIGLC and IF levels among patients with various GOLD grades. (A is serum FIGLC (κ and λ) level, and B is IF (such as TNF-α, IL-6, and IL-8) level.)

A comparison was made of serum levels of FIGLCs (κ and λ) and ICs (such as TNF-α, IL-6, and IL-8) among COPD patients with various GOLD grades (mild, moderate, severe, and very severe). The results revealed that as the COPD grade increased, FIGLCs and ICs serum levels showed an increasing trend (Figure 3).

### Correlation between IFs and pulmonary function indexes in COPD patients

**Figure 4:**
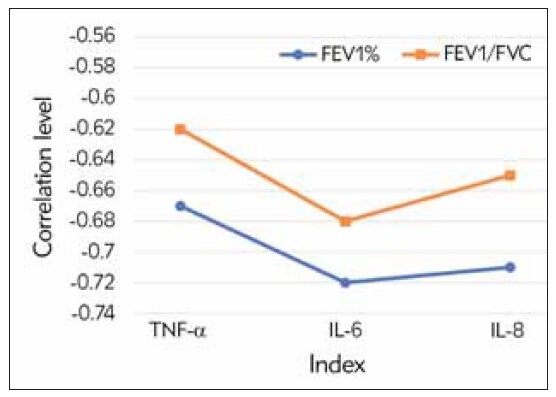
Correlation between IFs and pulmonary function indexes in COPD patients.

Further analysis was conducted on the correlation between serum IC levels (TNF-α, IL-6, IL-8) and lung function parameters (such as FEV1% predicted and FEV1/FVC ratio). The results indicated a negative correlation between IC levels and lung function parameters, indicating that higher levels of ICs were associated with lower lung function. In Figure 4, the correlation coefficient between TNF-α and FEV1% predicted was -0.67, indicating a negative correlation between the two variables, i.e., higher levels of TNF-α were associated with lower FEV1% predicted. Similarly, IL-6 and IL-8 were also negatively correlated with lung function parameters.

## Discussion

The mechanism by which serum FIGLCs affect pulmonary inflammation and serum levels of ICs in patients with COPD was explored. The results revealed that serum FIGLC levels were drastically higher in COPD patients versus controls. In addition, ICs (TNF-α, IL-6, IL-8) were negatively correlated with lung function parameters in COPD patients. Hence, it was verified that serum FIGLCs may play a critical role in lung inflammation in patients with COPD.

COPD is associated with chronic inflammation predominantly affecting the lung parenchyma and peripheral airways, resulting in irreversible and progressive airflow limitation. This inflammation is characterized by increased alveolar macrophages, neutrophils, T lymphocytes, and innate lymphoid cells [19]. Recent studies have highlighted the role of IgLC in COPD. Elevated levels of free IgLC have been observed in experimental and clinical COPD. These IgLC are produced directly by B cell-derived plasma cells and are found naturally in various body fluids. The researchers found that levels of IgLC were elevated in animals with emphysema and right heart enlargement caused by chronic cigarette smoke inhalation. In patients with moderate to severe COPD, increased serum levels of IgLC were found compared with controls [20]. The binding of IgLC to human neutrophils was shown to induce the chemokine IL-8 (CXCL8). This suggests that IgLC could play a role in the pulmonary inflammatory response observed in COPD patients [20].

Serum FIGLCs may exacerbate lung inflammation in patients with COPD by regulating the activity of inflammatory cells, promoting cytokine production, and increasing the release of inflammatory mediators [21]. The results suggested that serum FIGLC was drastically higher in COPD patients than healthy controls, suggesting that serum FIGLCs may be closely related to pulmonary inflammation in COPD. This finding is consistent with previous research and further confirms the significance of serum FIGLCs in COPD pathogenesis [22].

Comparing COPD patients with various GOLD grades, it was found that the levels of serum FIGLCs and ICs increased gradually with the severity of the disease. This indicates that the levels of serum FIGLCs and ICs are closely related to the severity of COPD and may serve as potential indicators for assessing the condition and predicting the disease progression [23]. A study by Tanimura et al. investigated the treatment and prognosis of patients with COPD. The researchers found that a low serum free light chain is a novel B-cell-associated biomarker for COPD exacerbations. Adaptive immunity via antibody production is important in preventing infections. Impaired antibody production is reported to be associated with an increased risk of exacerbations of COPD [24]. In addition, this trend also suggests that COPD patients with various severity levels may require targeted treatment strategies [25].

It was found that ICs (TNF-α, IL-6, IL-8) were negatively correlated with lung function indicators (FEV1% predicted, FEV1/FVC). This suggests that elevated levels of ICs may be related to the decline in lung function in those with COPD. This finding is consistent with other studies and further supports the crucial role of inflammation in the course of COPD [23]. These ICs may induce oxidative stress, cell infiltration, and tissue damage, impairing lung function in patients with COPD [26] [27] [28]. Therefore, interventions targeting these ICs may help delay lung function decline in COPD patients and improve their quality of life.

The results suggested that serum FIGLCs may act imperatively in the pulmonary inflammatory response of those with COPD, providing new clues for further understanding the pathophysiological mechanisms of COPD. In addition, serum FIGLC levels may serve as a potential indicator for assessing COPD severity and predicting disease progression. Future studies can further investigate the specific mechanisms of serum FIGLCs to guide targeted therapeutic strategies.

## Conclusion

This work found that the serum levels of FIGLCs in COPD patients were markedly superior to those in the healthy controls and were negatively correlated with levels of IFs and lung function indicators. In addition, the serum levels of FIGLCs were closely related to the severity of COPD. These findings suggest that FIGLCs may act imperatively in the pulmonary inflammatory response of patients with COPD and provide a potential indicator for evaluating and predicting COPD progression. However, further research is needed to elucidate the specific mechanisms of FIGLCs in the progression of COPD.

## Dodatak

### Conflict of interest statement

All the authors declare that they have no conflict of interest in this work.
